# Anakinra treatment in multisystemic inflammatory syndrome in children (MIS-C) associated with COVID-19

**DOI:** 10.3389/fped.2022.942455

**Published:** 2022-08-18

**Authors:** Şengül Çaǧlayan, Hafize Emine Sönmez, Gülçin Otar Yener, Esra Baǧlan, Kübra Öztürk, Kadir Ulu, Vafa Guliyeva, Demet Demirkol, Mustafa Çakan, Semanur Özdel, Hulya Bukulmez, Nuray Aktay Ayaz, Betül Sözeri

**Affiliations:** ^1^Department of Pediatric Rheumatology, Umraniye Training and Research Hospital, University of Health Sciences, Istanbul, Turkey; ^2^Department of Pediatric Rheumatology, Kocaeli University, Kocaeli, Turkey; ^3^Department of Pediatric Rheumatology, Şanlıurfa Research and Training Hospital, Şanlıurfa, Turkey; ^4^Department of Pediatric Rheumatology, Sami Ulus Research and Training Hospital, Ankara, Turkey; ^5^Department of Pediatric Rheumatology, Göztepe Research and Training Hospital, Istanbul Medeniyet University, Istanbul, Turkey; ^6^Department of Pediatric Rheumatology, Faculty of Medicine, Istanbul University, Istanbul, Turkey; ^7^Department of Pediatric Intensive Care Unit, Faculty of Medicine, Istanbul University, Istanbul, Turkey; ^8^Division of Pediatric Rheumatology, Department of Pediatrics, Metro Health Medical Center, Case Western Reserve University, Cleveland, OH, United States

**Keywords:** anakinra, COVID-19, multisystem inflammatory syndrome in children (MIS-C), treatment, refractory MIS-C

## Abstract

**Objective:**

The study aimed to report the efficacy and safety of anakinra treatment in patients with the refractory multisystemic inflammatory syndrome in children (MIS-C).

**Methods:**

This is a cross-sectional retrospective study consisting of pediatric patients diagnosed with MIS-C who were treated with anakinra.

**Results:**

Among the 378 patients diagnosed with MIS-C, 82 patients (21.6%) who were treated with anakinra were included in the study. The median age of patients was 115 (6-214) months. The median duration of hospitalization was 15 (6-42) days. Sixty patients (73.1%) were admitted to the pediatric intensive care unit. Patients were treated with a median dose of 2.7 mg/kg/day anakinra concomitant with IVIG and steroids. Intravenous anakinra was applied to 12 patients while 70 patients received it subcutaneously. Twenty-eight patients required high dose (4–10 mg/kg/day) anakinra. The median day of anakinra initiation was 2 (1-14) days and the median duration of anakinra use was 7 (1-41) days. No injection site reactions were observed while elevated transaminase levels were detected in 13 patients. Seventy-three patients (89.1%) were discharged without any sequela or morbidity. Seven patients (1.8%) died. Abnormal echocardiographic findings continued in two patients (2.4%) (coronary artery dilatation in one, low ejection fraction in one) at discharge and became normal on the 2^nd^ month.

**Conclusion:**

Based on the results of the study, anakinra was associated with clinical improvements and was safe for most patients with refractory MIS-C.

## Introduction

Multisystemic inflammatory syndrome in children (MIS-C) is a hyperinflammatory condition that has recently entered our lives after severe acute respiratory syndrome coronavirus 2 (SARS-CoV-2) infection and presents with Kawasaki-like disease and/or shock-like findings. The first 8 cases were reported from England (1). Subsequently, similar case reports began to come from all over the world.

A total of 6,851 MIS-C cases have been reported and 59 deaths have been observed since May 2020 (2). Most of the patients were negative for RT-PCR and positive for SARS-COV 2 antibodies. Patients present with various clinical findings including fever, rash, conjunctivitis, gastrointestinal (GI) symptoms, coagulopathy, cardiac involvement, and shock-like findings. As distinct from Kawasaki disease (KD), left ventricular dysfunction is the predominant cardiac feature in patients with MIS-C. Laboratory findings revealed an increased inflammatory response and some of the patients may progress to macrophage activation syndrome (MAS).

The underlying pathogenesis has not yet been fully elucidated and an abnormal immune response is blamed as the main factor in the pathogenesis of MIS-C. Various cytokines are thought to cause hyperinflammation, including, interleukin-1 (IL-1), interferon-gama (IFNγ), IL-18, IL-6, IL-8, and IL-10 (3, 4). While intravenous immunoglobulin (IVIG) and corticosteroids are used in the first line of treatment, anakinra is recommended as a therapeutic option in resistant cases (5). Anakinra is a recombinant IL-1 receptor antagonist. It acts as an anti-cytokine by inhibiting the binding of both IL-1-α and IL-1-β to IL-1 receptors. Anakinra is used to mitigate organ damage by hindering the cytokine storm that occurs in MIS-C. In this multicenter study, we aimed to present the data of patients using anakinra for refractory MIS-C.

## Methods

This is an international, cross-sectional study involving pediatric patients who were diagnosed with MIS-C and were treated with anakinra between May 2020 and December 2021, from six pediatric rheumatology centers Among the 378 patients diagnosed with MIS-C, 82 patients (21.6%) who were treated with anakinra were included in the study. The diagnosis of MIS-C was made according to WHO or CDC criteria (6, 7) and American College of Rheumatology Clinical Guidance (5) was used to guide the treatment. Anakinra dose has been adjusted according to the treating physician's decision. Clinical and laboratory findings, treatments, and outcomes were recorded retrospectively from medical charts and electronic files of the patients. Adverse events were defined as any undesirable or suspected reaction that occurred after anakinra treatment. Adverse events that prolonged hospital stay, or led to life-threatening conditions have been described as serious adverse events. Liver enzymes were accepted as increased if the elevation is ≥2 times the upper limit of normal. The definition of complete and incomplete KD was made according to American Heart Association (AHA) (8). The 2016 EULAR/ACR/PRINTO Classification Criteria for Macrophage Activation Syndrome was used for the classification of MAS patients (9).

Patients who had confusion, hypotensive course, sustained fever, hypoxia, multiple organ failures, and needed plasmapheresis, mechanical ventilation or ECMO were followed up in the intensive care unit. The study protocol was reviewed and approved by the Ethics Committee of the University of Health Sciences, Umraniye Training and Research Hospital (Approval No: B.10.1.TKH.4.34.H.GP.0.01/13) with the ethical principles laid down in the Declaration of Helsinki.

### Statistical analyses

The statistical analyses were made by using SPSS version 21.0 (SPSS, Inc., Chicago, Illinois). The variables were investigated using visual (histogram, probability plots) and analytic methods (Kolmogorov Smirnov/Shapiro–Wilk's test) to determine whether or not they are normally distributed. Descriptive analysis was presented using proportions, mean, standard deviation (SD), median, minimum (min), and maximum (max) values as appropriate.

## Results

### Baseline characteristics of patients

The study group consists of 82 patients with refractory MIS-C. Among them, 48 (58.5%) were male and 34 (41.5%) were female. The median age of patients was 115 (6–214) months. Sixty (73.2%) patients had a history of close contact with a symptomatic COVID-19 patient. Nasopharynx SARS-CoV-2 PCR was positive in 4 patients (4.9%) and the SARS-CoV-2 antibody test was positive in 78 patients (95.1%).

The most common clinical finding was fever, followed by cardiac, GI, and mucocutaneous features (Table 1). The median duration of fever before the diagnosis was 5 (1–30) days. Of these 82 patients, 41 (50%) had a Kawasaki-like phenotype and 34 (41.5%) fulfilled the classification of MAS.

**Table 1:** Clinical characteristics of MIS-C patients.

	**Patients (*n* = 82)**
Fever, *n* (%)	82 (100)
Mucocutaneous features	
.Polymorphous rash, *n* (%)	58 (70.7)
.Conjunctivitis, *n* (%)	51 (62.2)
.Oral changes, *n* (%)	29 (35.4)
.Extremity changes, *n* (%)	15 (18.3)
Cervical lymphadenopathy, *n* (%)	26 (31.7)
Mesenteric lymphadenopathy, *n* (%)	10 (12.2)
Hepatomegaly, *n* (%)	12 (14.6)
Splenomegaly, *n* (%)	10 (12.2)
Musculoskeletal features	
.Myalgia, *n* (%)	30 (36.6)
.Arthralgia, *n* (%)	28 (34.1)
Respiratory findings	
.Cough, *n* (%)	10 (12.2)
.Dyspnea, *n* (%)	17 (20.7)
Gastrointestinal findings	
.Abdominal pain, *n* (%)	52 (63.4)
.Nausea and vomiting, *n* (%)	28 (34.1)
.Peritonitis, *n* (%)	16 (19.5)
.Diarrhea, *n* (%)	21 (25.6)
.Bloody diarrhea, *n* (%)	5 (6.1)
Cardiac involvement	
.Hypotension, *n* (%)	56 (68.3)
.Tachycardia, *n* (%)	39 (47.6)
.Bradycardia, *n* (%)	8 (9.8)
.LV dysfunction or myocarditis, *n* (%)	34 (41.5)
.Mitral valve, *n* (%)	32 (39)
.Pericarditis, *n* (%)	14 (17.1)
.Coronary artery involvement, *n* (%)	4 (4.9)
Renal involvement, *n* (%)	3 (3.6)
Neurologic involvement	
.Headache, *n* (%)	18 (22)
.Loss of consciousness, *n* (%)	7 (8.5)

On the laboratory evaluation, lymphopenia was detected in 63 (76.8%) and thrombocytopenia in 33 (40.2%) patients. The C-reactive protein (CRP) levels were elevated in all patients while increased levels of erythrocyte sedimentation rate (ESR), ferritin, and procalcitonin were detected in 74 (90.2%), 43 (52.4%), and 58 (70.7%) patients, respectively. Five patients (6.1%) had elevated troponin-I levels, increased levels of pro-brain natriuretic peptide (BNP) were detected in 44 patients (53.6%), and D-dimer was increased in 69 patients (84.1%). Hypoalbuminemia and hyponatremia were detected in 59 (72%) and 55 (67.1%) patients, respectively (Table 2).

**Table 2:** Laboratory results of the patients' at the initiation of anakinra treatment.

**Complete blood count**	
WBC, mm^3^	9,530 (1,680–51,530)
Lymphocyte, mm^3^	875 (160–9,410)
NLR	8.5 (0.9–72)
Hemoglobin, g/dL	10.8 (3.1–14.7)
Platelet, mm^3^	170,500 (65,000–370,000)
**Inflammatory markers**	
CRP, mg/dL	16.1 (5.1–38.6)
ESR, mm/hr	47 (2–140)
Procalcitonin, ng/mL	11.3 (0.08–100)
Ferritin, ug/L	602 (127–13,437)
IL-6, pg/mL	390 (2–2,330)
**Cardiac markers**	
NT-pro-BNP, ng/L	5,550 (39–35,000)
Troponin-I, ng/L	0.01 (0–89.7)
**Coagulation parameters**	
D-dimer, μg/mL	3.95 (0.6–26.9)
Fibrinogen, g/L	543 (130–1,096)

*CRP, C-reactive protein; ESR, erythrocyte sedimentation rate; IL-6, interleukin-6; NLR, neutrophil-lymphocyte ratio; NT-pro-BNP, N-terminal prohormone brain natriuretic peptide; WBC, White blood count*.

### Treatments

Sixty (73.1%) patients required intensive care support within a median of 1.5 (1–15) days after hospitalization. The median duration of hospitalization was 15 (6–42) days and the median duration of stay in the intensive care unit was 5 (1–27) days. All patients received IVIG and corticosteroids. Thirty-one patients (31.8%) received pulsed methylprednisolone for three consecutive days (15–30 mg/kg/day; maximum dose: 1,000 mg/day) and then continued with a dosage of 2 mg/kg/day. The remaining 51 patients (68.2%) received 2 mg/kg daily dose of corticosteroids. In 12 patients (14.6%) IVIG treatment was completed to 2 g/kg in split doses. Twelve patients (14.6%) received intravenous and 70 patients (85.4%) received subcutaneous anakinra. Patients were treated with a median dose of 2.7 (2–10) mg/kg/day anakinra while the anakinra dose was increased to 4–10 mg/kg/day in 28 patients (34.1%). The median day of anakinra initiation was 2 (1–14) days and the median duration of anakinra use was 7 (1–41) days. Thirty-four patients (41.4%) received inotropic agents, and 19 patients (23.2%) received plasmapheresis. Prophylactic low-molecular-weight heparin (LMWH) (1 mg/kg/day) was given to 73 patients (89%).

### Outcome and safety

**Figure 1:**
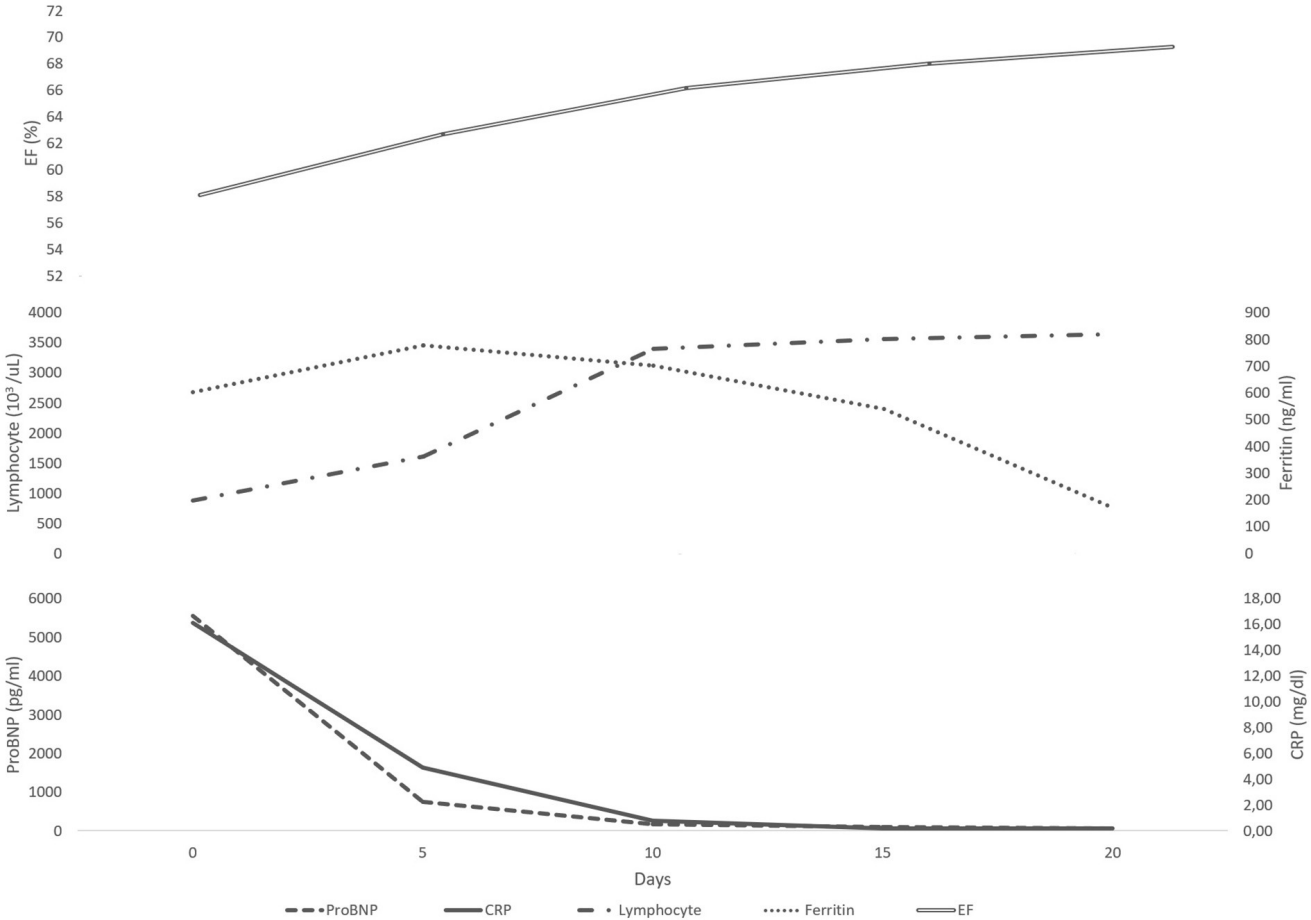
The daily variation of median laboratory values under anakinra treatment.

The median resolution day of the fever was 3 (1–10) days after anakinra treatment. The lymphocyte counts, BNP, CRP, and D-dimer values of the patients became normal on the median day of 5 (1–18), 8.5 (5–53), 10 (1–33), and 11.5 (4–20), respectively. The pattern of improvement in EF and laboratory parameters are shown in Figure 1. None of the patients experienced injection site reactions related to anakinra while elevated transaminase levels were observed in 13 patients (15.8%). Side effects that develop after the use of anakinra and concomitant treatments are shown in Table 3. Overall, after anakinra treatment, 63 patients achieved clinical improvement with anakinra without the need for additional treatments. Nineteen patients required plasmapheresis and 2 patients needed ECMO. Seven patients (8.5 %) died, five of which were due to severe MAS and two due to fulminant myocarditis. Abnormal echocardiographic findings continued in two patients (2.4%) (coronary artery dilatation in one, low ejection fraction in one) at discharge and became normal on the 2^nd^ month.

**Table 3:** Side effects observed under anakinra treatment.

**Patient number**	**AE**	**SAE**	**Concomitant treatments**
Patient 9	AST:123 U/L ALT:174 U/L		Ceftriaxone, noradrenaline, methylprednisolone, LMWH
Patient 26		Acute pancreatitis	Ceftriaxone, noradrenaline, methylprednisolone, LMWH
Patient 38	AST:95 U/L ALT:87 U/L		Ceftazidim, noradrenaline, methylprednisolone, LMWH
Patient 40	AST:142 U/L ALT:102 U/L		Vancomycin, meropenem, noradrenaline, methylprednisolone, LMWH
Patient 53	AST:183 U/L ALT:162 U/L		Cefotaxime, noradrenaline, methylprednisolone, LMWH
Patient 57	AST:137 U/L ALT:221 U/L		Cefotaxime, noradrenaline, methylprednisolone, LMWH
Patient 58	AST:115 U/L ALT:89 U/L		Vancomycin, meropenem, methylprednisolone, LMWH
Patient 59	AST:139 U/L ALT:346 U/L		Ceftriaxone, noradrenaline, methylprednisolone, LMWH
Patient 60	AST:183 U/L ALT:173 U/L		Vancomycin, meropenem, methylprednisolone, LMWH
Patient 63	AST:165 U/L ALT:83 U/L		Cefotaxime, noradrenaline, methylprednisolone, LMWH
Patient 68	AST:102 U/L ALT:92 U/L		Vancomycin, meropenem, noradrenaline, methylprednisolone, LMWH
Patient 70	AST:82 U/L ALT:94 U/L		Cefotaxime, noradrenaline, methylprednisolone, LMWH
Patient 75	AST:771 U/L ALT:859 U/L	Acute cholangitis	Ceftriaxone, noradrenaline, methylprednisolone, LMWH
Patient 78	AST:99 U/L ALT:160 U/L		Vancomycin, meropenem, noradrenaline, methylprednisolone, LMWH

*AE, Adverse event; SAE, serious adverse event; LMWH, low-molecular-weight heparin; AST, aspartate aminotransferase (N:5-34 U/L); ALT, alanine aminotransferase (N:0-41 U/L)*.

## Discussion

When MIS-C was first defined, IVIG was announced as the main treatment for this new disease because of its similar clinical findings with KD. However, after the clarification of cytokine storm in the pathogenesis of MIS-C, anakinra came to the fore as an alternative treatment in IVIG-resistant cases. Anakinra, a recombinant interleukin (IL)-1 receptor antagonist, is a safely preferred agent in children with MAS. But, the efficacy and safety of anakinra in the treatment of MIS-C is still unclear due to the lack of large controlled clinical trials. We have observed that it was a successful treatment modality in most of the MIS-C patients.

As MIS-C is an emerging phenomenon, new approaches were needed to guide healthcare providers when treating patients. For this purpose, the ACR announced a recommendation set for the management of MIS-C on May 22, 2020 (5). Subsequently, this recommendation set was updated (10). According to the latest version, IVIG and/or corticosteroids are considered first-tier agents and in the presence of a refractory course, high-dose anakinra (>4 mg/kg/day IV or SC) should be initiated (10). The use of anakinra for pediatric inflammatory disease is rapidly expanding with its rapid effect and short half-life. Furthermore, it has a good safety profile even in high doses. In our study, the median dose of anakinra was 2.7 (2–10) mg/kg/day. Depending on the difficulties in the recruitment of anakinra and the immediate treatment needs of the patients, sometimes anakinra vials were shared with more than one patient. Since clinical improvement was observed in the patients, low-dose treatment was continued. All of the patients with poor, rapidly deteriorating general conditions received the recommended high doses of anakinra Although routine administration of anakinra is SC, there are also studies on its IV use. Phadke et al. (11) showed the safety and efficacy of IV anakinra in the treatment of MAS. Cavalli et al. (12) showed that high-dose (10 mg/kg/day) IV anakinra reduced the need for invasive mechanical ventilation or death in patients with hyper-inflammation due to COVID-19. Herein, we presented 82 pediatric MIS-C patients who were treated with a 2.7 mg/kg/day median dose of anakinra while high-dose (>4 mg/kg) anakinra was used in 28 patients (34.1%). All of them were IVIG and corticosteroid resistant and had a severe disease course. Of 82 patients, IV anakinra was used in 12 patients (14.6%). In the presence of thrombocytopenia, subcutaneous edema, or shock, IV administration instead of SC anakinra may be an alternative. In the present study, the patients receiving IV anakinra had left ventricular dysfunction and no anaphylactic reaction was observed. Continuous IV infusion of anakinra provided clinical improvement in 4/5 patients with MAS (13). Furthermore, recently, the efficacy and safety of IV anakinra in patients with non-familial haemophagocytic lymphohistiocytosis (HLH) even in extremely high doses (48 mg/kg/day) has been reported (14). Intravenous anakinra could be an option in selected patients and appears to be well tolerated at high doses.

The studies focusing on anakinra in refractory MIS-C patients are limited. Bhat et al. (15) showed successful outcomes for two refractory MIS-C patients treated with anakinra. Besides anakinra, other biologic drugs such as tocilizumab and infliximab were announced as an alternative option in refractory patients. Compared with other biologic drugs, anakinra's short half-life makes it more suitable. It is well known that anakinra is a safe drug for patients with severe sepsis. Çelikel et al. (16) evaluated the role of biological agents in the treatment of MIS-C. They prescribed anakinra to 23 patients with MIS-C and showed a significant resolution in laboratory parameters (16). Furthermore, successful results with anakinra in patients with the multisystem inflammatory syndrome in adults were published (17).

Injection site reaction is the most common side effect of anakinra treatment (18). However, no injection site reaction was observed in the present study. Cavalli et al. (12) reported elevated transaminase levels (more than three times the upper limit of normal) in 13% of patients with hyper-inflammation due to COVID-19 receiving anakinra. Correspondingly, elevated transaminase levels were observed in 15.8% (*n* = 13) of our patients. However, it is difficult to clearly state whether this was an adverse effect or was related to the disease course.

According to adult studies, anakinra reduces the need for mechanical ventilation, length of stay in the hospital and intensive care unit, and mortality in patients with COVID-19 (19, 20). But, unfortunately, despite intensive treatment, the mortality rate of MIS-C is reported to range between 1.2 and 1.7% (21, 22). In the present study, seven patients died despite all treatments. The mortality rate in our total MIS-C patients was 1.8%, which is similar to the literature, but the mortality rate in patients using anakinra was 8.5%. Of those who died, one had systemic juvenile idiopathic arthritis and one had Kostmann disease. The other five patients did not have any known comorbidity. Two patients died due to fulminant myocarditis, and five patients died due to multiorgan failure after MAS. These patients had a severe and rapid disease course from the first admission to the hospital. Pulse corticosteroids, IVIG, and anakinra treatments were applied to all of the patients, five patients underwent plasmapheresis, and ECMO was used in two patients. However, despite all treatments, these patients died. The main limitation of our study is its retrospective design with the absence of a control or placebo group. However, the study supports that anakinra may be an effective treatment option in refractory MIS-C patients.

In conclusion, since the release of interleukin-1 is a key role in the pathogenesis of cytokine storm syndrome, anakinra seems to be the preferred agent in MIS-C patients. According to the results of our study, anakinra could be a successful treatment modality in IVIG and corticosteroid unresponsive, refractory MIS-C cases. Compared to other biologic agents, short half-life of anakinra brings the drug to the stage when quick treatment decisions regarding the patient's treatment is required.

## Data availability statement

The original contributions presented in the study are included in the article/supplementary material, further inquiries can be directed to the corresponding author/s.

## Author contributions

BS and ŞÇ conceptualized and designed the study, drafted the initial manuscript, and had full access to all the data in the study. All authors conducted the data analyses, drafted the initial manuscript, had full access to all the data in the study, reviewed and revised the manuscript, and approved the final version of the manuscript.

## Conflict of interest

The authors declare that the research was conducted in the absence of any commercial or financial relationships that could be construed as a potential conflict of interest.

## Publisher's note

All claims expressed in this article are solely those of the authors and do not necessarily represent those of their affiliated organizations, or those of the publisher, the editors and the reviewers. Any product that may be evaluated in this article, or claim that may be made by its manufacturer, is not guaranteed or endorsed by the publisher.
